# Development of a Novel Method to Detect AAV Vector Integration

**DOI:** 10.3390/v18030315

**Published:** 2026-03-03

**Authors:** Junping Zhang, Thao Thi Dang, Tsai-Yu Lin, Xiangping Yu, Danilo Pellin, Jiahe Tian, Olga Simmons, Emma Kou, Kenneth Cornetta, Weidong Xiao

**Affiliations:** 1 Department of Pediatrics, Herman B Wells Center for Pediatric Research, Indiana University School of Medicine, Indianapolis, IN 46202, USA; zhanju@iu.edu (J.Z.); jt828@cornell.edu (J.T.); 2 Department of Medical and Molecular Genetics, Indiana University School of Medicine, Indianapolis, IN 46202, USAlinty@iu.edu (T.-Y.L.);; 3 Division of Hematology/Oncology, Boston Children’s Hospital, Harvard Medical School, Boston, MA 02115, USA; 4 Department of Pediatric Oncology, Dana-Farber Cancer Institute, Harvard Medical School, Boston, MA 02115, USA; 5 Department of Computer Science, Cornell University, Ithaca, NY 14853, USA

**Keywords:** AAV vector, AAV integration, CRISPR-Cas9, nanopore, sequencing, hybridization capture

## Abstract

AAV integration has become an important safety consideration in gene therapy. However, accurately determining integration sites remains challenging due to biases introduced by library preparation methods, sequencing technologies, and bioinformatic pipelines. In this study, we developed a PCR-free amplification based on a CRISPR-Cas9 cleavage strategy for AAV DNA that overcomes the limitations of PCR amplification imposed by the ITR structure. When combined with long-read nanopore sequencing, this CRISPR-Cas9-based workflow preserves native AAV integration states and enables unbiased detection of integration junctions. We used AAV-transduced HeLa single-cell clones to evaluate the performance of this approach. To confirm integration site identification, AAV integration junctions were also detected using a probe hybridization capture strategy followed by Illumina short-read sequencing. Integration junctions identified by both methods were further confirmed by PCR. The results showed strong consistency between the two approaches in accurately identifying AAV integration sites in each clone. Overall, these findings demonstrate that the CRISPR-Cas9-enabled, PCR-free long-read sequencing workflow provides a promising tool for characterizing AAV integration events.

## 1. Introduction

Adeno-associated virus (AAV) vectors are being actively developed for a variety of diseases, with clinical products approved for congenital blindness, spinal muscular atrophy, aromatic L-amino acid decarboxylase deficiency, and hemophilia A and B [1]. AAV vectors have also been utilized to deliver guide RNAs or editing enzymes for gene-editing applications [2,3]. The majority of AAV vectors enter and persist as circular concatemeric episomes in target cells [4], while a minority are integrated into the target cell genome, a process mediated by cellular DNA repair pathways [4,5]. Integration occurs at sites of double-stranded breaks with a propensity for transcriptionally active areas, CpG islands, and transcription start sites [5,6,7,8]. The AAV vector sequence and the impact of partial vector genomes in vector products appear to increase the rate of integration [9,10]. Moreover, insertions appear to have a high rate of deletion, additional, and rearrangements [8,11].

To date, there has been no report of AAV-mediated insertional oncogenesis in humans, but AAV integration has led to insertional oncogenesis in a limited number of murine models of AAV gene therapy [12]. AAV vectors have also led to clonal cell expansion in a canine model of hemophilia [13]. Hepatocellular carcinoma and adenomas have been associated with AAV vector trials, with initial analysis suggesting they are unrelated to the gene therapy, but investigations are ongoing [12]. Available data suggest the risk of insertional mutagenesis depends on the intrinsic properties of the vector and the physiological and developmental state of the target cell [12,14].

For integrating vectors such as those based on murine and human retroviruses, robust methods are available for amplifying, sequencing, and analyzing vector integrations [15,16,17]. These methods take advantage of the integrase-mediated insertion, which preserves the retrovirus genome with high accuracy. In contrast, cellular DNA repair pathways integrating AAV concatemeric episomes are associated with mutations in both the vector and cellular DNA. Moreover, the high GC content and palindromic nature of AAV ITR have been a major obstacle for sequencing AAV genomes. Several approaches to AAV insertion site analysis have been studied. An important initial study used a shuttle vector system [8], with later studies modifying the LAM-PCR or INSPIIRED methods used for retroviral insertion site detection [7,11,13,16]. These methods use restriction enzymes or sonication to generate DNA fragments onto which DNA linkers are added. PCR amplification is then performed using primers targeting the vector and ligated sequence-generating fragment containing the genomic–vector junction. For AAV vector detection, multiple primers targeting the vector sequence are required due to the frequent deletions in the vector sequence. Initial sequencing utilized the Illumina platform, while more recent studies have utilized targeted enrichment sequencing [18,19] or long-read sequencing using the PacBio or Nanopore methods [20,21,22,23,24]. Bioinformatic analysis has utilized a variety of methods, but PCR amplification and sampling limitations lead to bias, especially when attempting to correlate sequencing reads with clonal cell expansion [25]. While insertion site detection methods have advanced, continued improvements are still needed.

CRISPR-Cas9 has become a powerful gene-editing technology that uses a guide RNA to direct the Cas9 nuclease to a specific DNA sequence, enabling precise cleavage and modification of the target site. Beyond its wide use in genome editing, CRISPR-Cas9 has also emerged as a valuable tool for targeted DNA enrichment. Several amplification-free CRISPR-based enrichment strategies have recently been integrated with next-generation sequencing platforms, such as Cas9/nanopore sequencing, Cas9/metagenomic deep sequencing (HiSeq 4000), and SpCas9 or FnCpf1 coupled with Illumina MiSeq systems, among others [26,27,28,29]. Cas9-guided cleavage enables selective isolation of long genomic segments without PCR amplification, thereby minimizing amplification bias and facilitating the analysis of structurally complex or repetitive loci. These approaches can capture intact genomic fragments spanning tens of kilobases [30], making them particularly well suited for long-read sequencing technologies. As a result, CRISPR-Cas9 enrichment has expanded the capacity of genomic analyses, supporting applications such as fusion gene detection [28], structural variant characterization [28,30], low-frequency mutation analysis (<0.001) [31], viral integration mapping [29], and characterization of gene therapy vectors [29].

In this study, we employ a CRISPR-Cas9-based workflow in which fragmented DNA is dephosphorylated prior to Cas9-mediated cleavage to facilitate preferential adaptor ligation at Cas9-generated DNA ends. This design enables efficient enrichment of AAV vector sequences while preserving the native junctions between integrated AAV genomes and host DNA. When combined with long-read nanopore sequencing, this approach supports high-resolution mapping of AAV integration events and more accurate reconstruction of their structural configurations.

## 2. Materials and Methods

### 2.1. Virus Transduction and Cell Line Production

HeLa cells (ATCC, CCL-2, Manassas, VA, USA) were used to generate single-cell clones containing the single-stranded AAV2-CAG-eGFP vector. HeLa cells were cultured in high-glucose DMEM (Cytiva, SH30243.FS, Marlborough, MA, USA) supplemented with 10% FBS (Corning, 35-015-CV, Corning, NY, USA) and 1% penicillin–streptomycin (100 U/mL and 100 μg/mL; Gibco, 15140122, Carlsbad, CA, USA) at 37 °C with 5% CO_2_. Cells (1 × 10^5^ per well) were seeded in 6-well plates, incubated overnight, and treated with or without 50 µM of bleomycin sulfate (Selleck Chemicals, S1214-10MG, Houston, TX, USA) for 2 h. After washing with DMEM containing 2% FBS, cells were transduced with AAV2-CAG-GFP particles (Addgene, 37825-AAV2, Watertown, MA, USA; 7 × 10^12^ vector genomes per mL (vg/mL) at 5 × 10^4^ vg/cell for 24 h). The medium was replaced, and cells were passaged every 4 days. At 13 days post-transduction, the percentage of GFP^+^ singlet cells was calculated based on gating with non-transduced cells, and the eGFP^+^ cells were sorted (BD Biosciences, FACSAria Fusion, San Jose, CA, USA). After an additional 4 weeks of culture, single GFP^+^ cells were sorted into a well of 96-well plates. The single-cell clones were expanded and passaged for an additional 6 weeks. Seven HeLa-AAV2-eGFP single-cell clones were evaluated for GFP expression by flow cytometry (BD Biosciences, LSRFortessa, San Jose, CA, USA) and analyzed using FlowJo v11 software (BD Biosciences, San Jose, CA, USA). The average vector copy number per cell (VCN) was determined by droplet digital polymerase chain reaction (ddPCR).

### 2.2. DNA Library Preparation for Long-Read Nanopore Sequencing

Genomic DNA was extracted using the GeneJET Genomic DNA purification kit (Thermo Fisher Scientific, 332659, Waltham, MA, USA) and sheared into approximately 10 kb DNA fragments by a g-TUBE (Covaris, 520079, Woburn, MA, USA). The DNA fragments were dephosphorylated by FastAP™ thermosensitive alkaline phosphatase (Thermo Fisher Scientific, EF0651, Waltham, MA, USA) and purified using a gel column (GeneJET Gel Extraction kit, Thermo Fisher Scientific, K0691, , Waltham, MA, USA). The purified DNA was cleaved in the reaction of sgRNAs and Cas9 nuclease mixture, in which the sgRNAs targeted the AAV2-CAG-e GFP vector (GenScript Biotech, L00689-30, Piscataway, NJ, USA). The list of sgRNAs targeting the AAV vector sequence is provided in the Supplementary Data, Table S2. Following the Cas9 nuclease cleavage, DNA was purified by a gel column for subsequent library preparation, and dA-tailing for the DNA library was performed by the Klenow fragment (3′ → 5′ exo-) (NEB, M0212S, Ipswich, MA, USA). The samples were purified by AMPure XP beads for native adaptor ligation following the manufacturer’s instructions provided by the Native Barcoding Kit 24 V14 (Oxford Nanopore Technologies, SQK-NBD114.24, Oxford, UK). Sequencing was performed on a PromethION platform using R10.4.1 flow cells (Oxford Nanopore Technologies, FLO-MIN114, Oxford, UK) at the Indiana University School of Medicine Center for Medical Genomics Service Core (IUSM-CMG).

### 2.3. DNA Library Preparation for Short-Read Illumina Sequencing

Genomic DNA (gDNA) samples were extracted using the DNeasy Blood & Tissue Kit (QIAGEN, 69504, Germantown, MD, USA) following the manufacturer’s protocol. An amount of 1 µg of DNA was fragmented by sonication using the Covaris ME220 sonication system with a 70 Watts peak power, a 20% duty factor, and 1000 cycles/burst at 20 °C for 110 s for a total run time of 5 min and 30 s and purified using 1.5X SPRISelect beads (Beckman Coulter, B23317, Brea, CA, USA) to obtain gDNA fragments with a size of approximately 250 bp. Purified gDNA fragments were used for DNA library preparation using the xGEN DNA Library Prep MC Kit (Integrated DNA Technologies (IDT), 10009861, Coralville, IA, USA) following the manufacturer’s procedure with some modifications. In brief, gDNA fragments were generated by end prep, adapter ligation, and cleanup by 1.2X SPRISelect beads, and the PCR amplification using xGen UDI primer pairs (IDT, 10005975, San Jose, CA, USA). The PCR program was as follows: 1. 98 °C for 2 minutes, 2. 14 cycles at 98 °C for 20 seconds, 60 °C for 30 s, and 72 °C for 30 s, and 3. 72 °C for 1 minute. Post-PCR cleanup was performed using 1.5X SPRISelect beads.

Prepared library fragments were then separated from off-target fragments using the xGen Hybridization Capture protocol from IDT with modifications. First, xGen™ universal blockers (IDT, 1075474, San Jose, CA, USA) were mixed with prepared library fragments to prevent adapter-to-adapter hybridization. Blocked library fragments were then subjected to hybridization capture using the xGen™ Custom Hyb Panel-Accel Probe for 16 h. The probe consisted of 71 sequences, each being 120 nucleotides in length, designed by IDT NGS Design to capture a 2928 bp DNA region spanning from ITR to ITR, based on the AAV vector sequence pAAV-CAG-eGFP (Addgene, 37825, Watertown, MA, USA). Next, the probe and fragment were then purified using streptavidin-coated magnetic beads, followed by washing steps using the xGen™ Hybridization and Wash Kit (IDT, 1080577, San Jose, CA, USA). The library containing only AAV target fragments was amplified by PCR following the manufacturer’s protocol and purified using 1.5X AMPure XP beads (Beckman Coulter, A63881, Indianapolis, IN, USA). The purified PCR product quality was determined by Agilent Tapestation using a TapeStation D5000 DNA ScreenTape Analysis at IUSM-CMG. A total of 8 prepared DNA libraries, which consisted of seven Hela-AAV2-eGFP single-cell clones and an untransduced HeLa cell negative control sample, were sequenced using NextSeq 1000/2000 P1 reagents (300 cycles) on the NextSeq 2000 (Illumina) at IUSM-CMG.

### 2.4. Bioinformatic Identification of AAV Integration Sites from Long-Read Sequencing

Raw signal data were base-called with Guppy v4.3.0 using the dna_r10.4.1_e8.2_400bps_hac model. Demultiplexing and adapter trimming were performed with the default MinKNOW workflow.

To jointly align host and vector sequences, we built a hybrid reference comprising the GRCh38 primary assembly and the complete AAV vector genome used in this study. Base-called reads were aligned to the hybrid reference using minimap2 v2.26 with the Nanopore long-read preset (-ax map-ont) [32]; supplementary alignments and split mappings were retained to capture chimeric host–AAV junctions. The resulting BAM files were coordinate-sorted and indexed with samtools v1.18 [33], and read-level QC summaries were generated using NanoStat for downstream analysis [34]. AAV integration events were identified with long-read SV callers configured to report the list of supporting read names per each breakpoint [35,36]. We selected candidate events in which one breakpoint mapped to the AAV vector and the partner breakpoint mapped to the human genome, corresponding to putative host–vector junctions. The event with the highest supporting reads was designated the baseline integration site for that clone. Because individual Nanopore reads span hundreds to thousands of base pairs across the host–vector junction, this analysis provided a single-molecule resolution of the local integration structure, allowing us to resolve small indels at the breakpoint and structural alterations within the vector sequence. All primary integration events and their supporting chimeric reads were visualized in IGV [37] using a custom chimeric reference (±5 kb of flanking human sequence with the AAV2-eGFP cassette inserted at the junction).

### 2.5. Bioinformatic Identification of AAV Integration Sites from Short-Read Sequencing

Short-read paired-end sequencing data generated with Illumina were processed using a custom computational workflow designed to identify AAV integration sites. A hybrid reference genome was created by concatenating the full human GRCh38 (hg38) assembly with the complete AAV vector sequence used in this study. This hybrid reference was indexed with minimap2, selected for its sensitivity to split-read structures and its ability to accurately align chimeric fragments that contain both vector- and host-derived sequences. Raw R1 and R2 FASTQ files were aligned to the hybrid genome using minimap2 with the short-read preset sr, producing alignment files that retained soft-clipped bases and supplementary alignments required for the detection of vector-to-genome junctions.

Reads were considered candidates for integration site analysis when they contained alignments to both the human genome and the AAV vector. Chimeric reads were extracted directly from sorted BAM files, the complete nucleotide sequence was retrieved from the original R1 FASTQ file using seqkit, converted to FASTA format, and refined through local alignment with BLAT.

Candidate integration site coordinates were derived using a structured post-processing procedure implemented in R with the data.table package. All BLAT output files in PSL format were imported and processed to evaluate, for each read, the best-supported AAV alignment and the best-supported human alignment. For each alignment block, a match score was computed as the proportion of matched bases relative to the aligned span. Only alignment blocks exceeding user-defined thresholds (>35 nt) for matching bases for both AAV and human segments were retained.

To reduce false-positive integration events, accepted reads were required to show consistent strand orientation and local proximity between vector and human alignments along the read. The AAV-aligned and human-aligned segments were required to fall within a small coordinate window around each other, ensuring that the observed chimeric structure reflected a contiguous junction.

For each accepted read, the integration site coordinate was computed directly from the refined human alignment and depended on the orientation of the vector-derived segment. The workflow returned the chromosome, the genomic coordinate of the junction, the integration direction, and detailed read-level statistics describing the AAV and human alignments. Results were aggregated across all PSL files to produce both a complete annotation table and a summary table reporting the number of supporting reads for each unique genomic coordinate.

### 2.6. Validation of AAV Integration Sites

Integration sites were confirmed by nested PCR using site-specific primers and gDNA extracted from HeLa-AAV2-eGFP clones. Nested PCR 2 was performed only when a specific product was not detected in the first amplification round. The PCR primers are listed in Supplemental Table S1. The PCR program was as follows: 1. 98 °C for 30 s, 2. 29 cycles at 98 °C for 30 seconds, 60 °C for 30 s, and 72 °C for 30 s, and 3. 72 °C for 30 s. PCR products were purified (Thermo Fisher Scientific, K0691, Waltham, MA, USA), sequenced (Plasmidsaurus, South San Francisco, CA, USA), and aligned using SnapGene v8.1 (SnapGene, Boston, MA, USA).

### 2.7. Droplet Digital PCR (ddPCR)

Genomic DNA was extracted using the Gentra Puregene DNA Isolation Kit (QIAGEN, 158389, Germantown, MD, USA). ddPCR was performed according to the manufacturer’s instructions for the QX200 droplet digital PCR system (Bio-Rad, Hercules, CA, USA), as previously described [17], with 0.05 µg of DNA input per reaction. Reactions were analyzed using the QX Manager v1.2 Regulatory Edition software (Bio-Rad, Hercules, CA, USA).

## 3. Results

### 3.1. Generation and Characterization of HeLa-AAV2-eGFP Single-Cell Clones for AAV Genome Integration Assay

**Figure 1:**
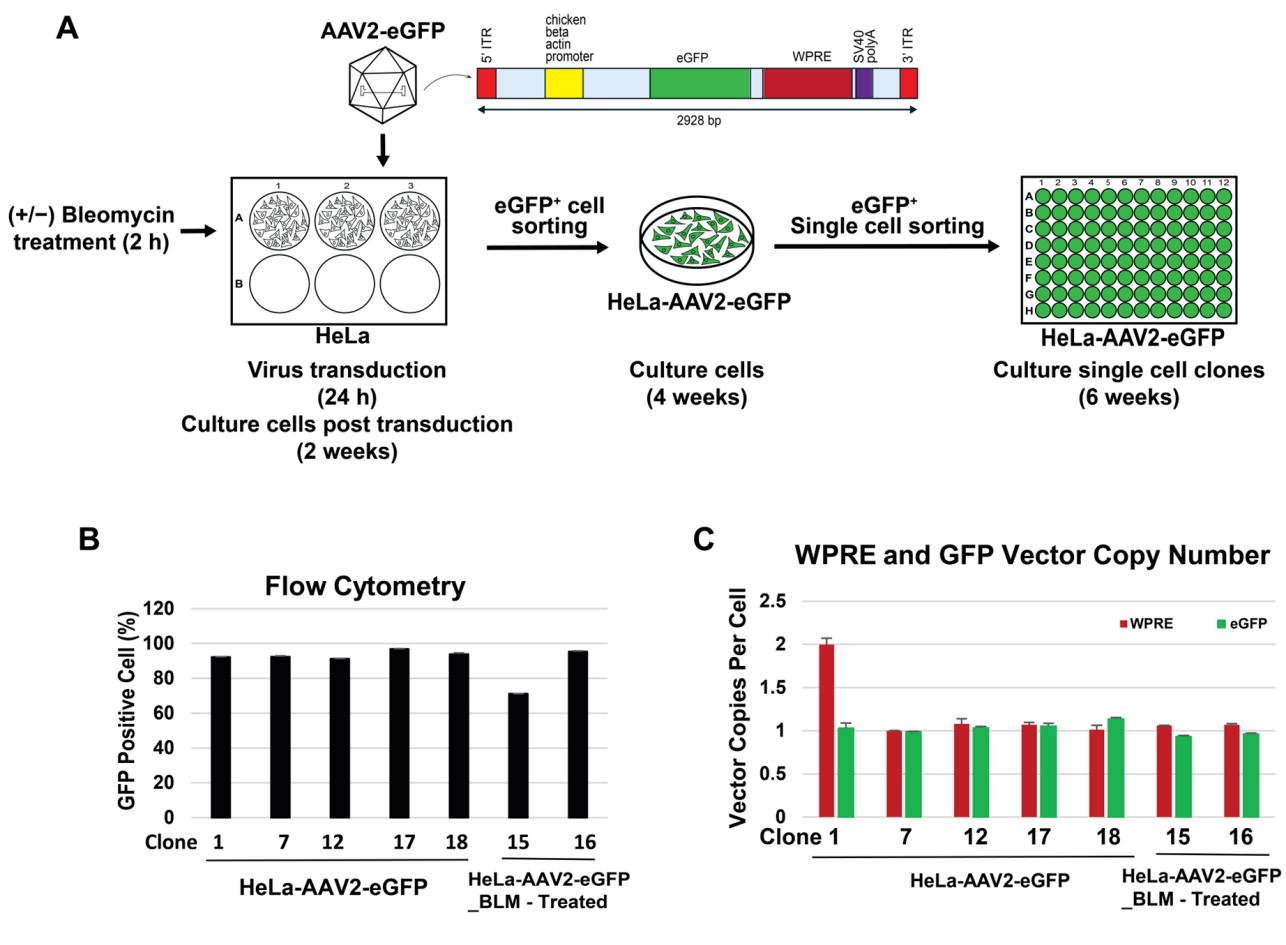
Generation and characterization of HeLa-AAV2-eGFP single-cell clones for AAV genome integration assay. (**A**) Schematic overview of the workflow used to establish HeLa-AAV2-eGFP single-cell clones. HeLa cells were treated with or without 50 uM of bleomycin for 2 h prior to AAV2-eGFP transduction at 50,000 vg/cell. Following 24 h of transduction and two weeks of culture, eGFP^+^ cells were enriched by flow cytometry and expanded for four weeks. A second round of single-cell sorting was used to obtain individual eGFP^+^ clones, which were cultured for an additional six weeks, after which the single-cell clones were analyzed by (**B**) percentage of eGFP expression by flow cytometry and (**C**) vector copy number by ddPCR using WPRE (red) and eGFP (green) primers and probes.

To establish AAV2 integration single-cell clones for genome integration assay, HeLa cells were pretreated for 2 h with or without the DNA-damaging agent 50 μM bleomycin (BLM). The cells were then transduced with the AAV2-eGFP vector at 50,000 vg/cell for 24 h. Two weeks post-transduction culture, eGFP-positive (eGFP^+^) cells were enriched by flow cytometry sorting and further expanded for an additional four weeks, at which time the percentage of eGFP^+^ cells and the vector copy numbers (VCNs) of WPRE and eGFP were quantified (Figure S1A,B). As predicted, BLM pretreatment significantly increased the proportion of eGFP^+^ cells (76.8%) compared with untreated cells (25.1%). The population of BLM-treated cells showed a WPRE and eGFP VCN of 1.1 and 1.2 copies per cell, respectively, compared with 0.35 copies per cell in untreated cells. The two populations of cells were then subjected to single-cell sorting, and eGFP+ clones were cultured for an additional six weeks. Five clones from the BLM-untreated and 2 clones from the BLM-treated condition were selected for VCN analysis using WPRE and eGFP ddPCR assays (Figure 1B,C). Flow cytometry analysis demonstrated that the percentage of eGFP+ cells in all clones remained high and stable, indicating long-term retention and expression of the eGFP transgene. VCN analysis for both WPRE and eGFP targets showed that most single-cell clones contained one vector copy per cell, with high consistency between WPRE and eGFP sequences. Clone 1 exhibited two copies of WPRE and one copy of eGFP per cell. These differences are consistent with expected variability arising from integration site structure, partial vector rearrangements, or differential retention of vector elements. Together, these findings demonstrate that BLM pretreatment enhances AAV transduction efficiency via the DNA strand break and repair mechanism. Moreover, the HeLa-AAV2-eGFP single-cell clones display stable and robust eGFP expression and contain single-copy vector genome insertions, providing well-characterized cellular models suitable for AAV genome integration assay development.

### 3.2. Overview of Target Enrichment Methods for Genome Integration Assay

**Figure 2:**
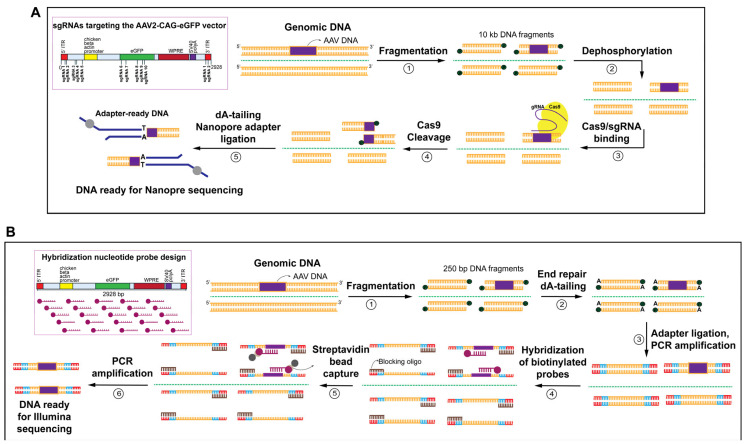
Schematic overview of target enrichment methods by PCR-free amplification based on CRISPR-Cas9 cleavage and probe hybridization capture. (**A**) PCR-free amplification based on CRISPR-Cas9 cleavage target enrichment for long-read Nanopore sequencing. (1) Shear genomic DNA (~10 Kb) and clean up with gel column, (2) dephosphorylation of DNA fragments, (3) prepare Cas9/sgRNA-binding reaction, (4) Cas9-gRNA cleavage of DNA, and (5) adaptor and barcode ligation for Nanopore sequencing. (**B**) Probe hybridization capture workflow for short-read Illumina sequencing. (1) Genomic DNA is fragmented (~250 bp), (2) end-repaired and dA-tailing, (3) adapters are ligated, followed by indexing PCR amplification, (4) blocking oligonucleotides remove nonspecific hybridization and biotinylated probes complementary to target regions hybridize to the prepared DNA fragments, (5) streptavidin-coated magnetic beads capture probe-bound fragments, and (6) PCR amplification and sequencing in Illumina platform.

We developed a novel PCR-free amplification based on CRISPR-Cas9 cleavage target enrichment compatible with long-read nanopore sequencing (Figure 2A and Figure S2A). In this approach, high-molecular-weight genomic DNA (10 kb) was fragmented and dephosphorylated prior to site-specific cleavage by the Cas9-gRNA complex. The resulting long fragments are compatible with Nanopore sequencing without amplification, completing target enrichment library preparation in approximately five hours. We compared this workflow with a probe hybridization capture approach combined with short-read Illumina sequencing (Figure 2B and Figure S2B). Unlike the CRISPR-Cas9-based approach, the hybridization-based approach uses biotinylated oligonucleotide probes that hybridize to adapter-ligated short DNA fragments (250 bp). Captured targets are isolated via streptavidin-coated magnetic beads and amplified by PCR to produce sequencing-ready libraries. Although this method provides high capture specificity and scalability for short-read sequencing, it requires more than 20 h of preparation, introduces amplification bias, and cannot recover long-range structural information or preserve native DNA modifications. The steps for DNA library preparation of the two approaches were described in detail in the Section 2.

Collectively, PCR-free amplification based on CRISPR-Cas9 cleavage target enrichment is amplification-free and uses several guide RNAs to cover and improve detection of incomplete genomes, whereas probe hybridization capture depends on biotinylated probes to capture the target region. Our novel CRISPR-Cas9-based workflow, thus, enables amplification-free, long-read sequencing that retains epigenetic and structural integrity, offering a rapid and streamlined alternative to conventional short-read capture methods.

### 3.3. Validation of AAV Integration Sites in HeLa-AVV2-eGFP Single-Cell Clones

**Figure 3:**
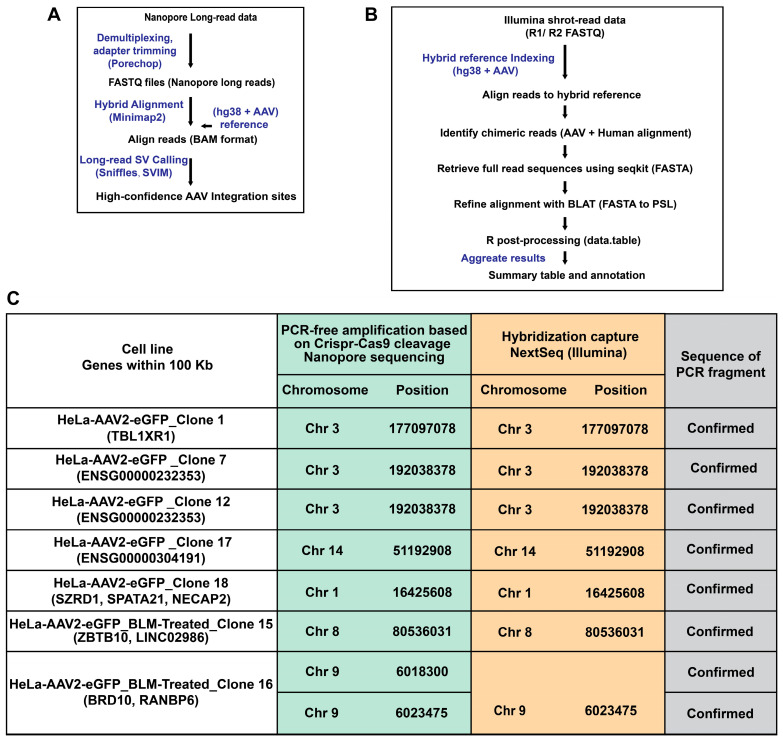
Validation of AAV integration sites detected by PCR-free CRISPR-Cas9 Nanopore sequencing and hybridization-based Illumina sequencing. (**A**,**B**) Schematic overview of the bioinformatic pipeline used to identify AAV2-eGFP integration sites in HeLa-AAV2-eGFP single-cell clones from Nanopore long-read data (**A**) and Illumina short-read data (**B**). (**C**) Comparative analysis of integration coordinates detected by PCR-free amplification based on CRISPR-Cas9 cleavage and hybridization capture. Integration sites were mapped to the indicated chromosomes at the same breakpoint positions confirmed between both methods.

Seven HeLa-AAV2-eGFP single-cell clones were characterized and shown to contain a single copy of the AAV2-eGFP transgene (Figure 1). We then sought to determine the ability of the CRISPR-Cas9-based method to detect the AAV vector integration site and compare that to data obtained using the hybridization capture method using separate analysis pipelines appropriate for each method. For the CRISPR-Cas9-based library, nanopore reads were demultiplexed and adapter-trimmed, and the resulting FASTQ files were aligned with minimap2 to a hybrid reference comprising the human GRCh38 genome and the AAV2-eGFP vector sequences. Structural variants and vector–host junctions were then called from the alignment BAM files using the long-read SV callers (Sniffles and SVIM). High-confidence AAV2-eGFP integration sites were used for downstream genomic annotation and visualization (Figure 3A). For the probe hybridization capture library, first, the AAV vector sequence was combined with the human GRCh38 reference to generate a hybrid alignment genome. Short reads (R1 and R2) from Illumina sequencing were aligned to this indexed hybrid reference using minimap2. Reads containing both human and AAV vector sequences were isolated, converted to FASTA format and refined through local alignment with BLAT. The resulting PSL files were processed in R to parse alignment blocks and apply scoring and filtering criteria to determine precise vector–host junction coordinates. The finalized dataset is summarized in a full annotation table listing read-level integration information and a summary table reporting unique genomic insertion sites (Figure 3B).

**Figure 4:**
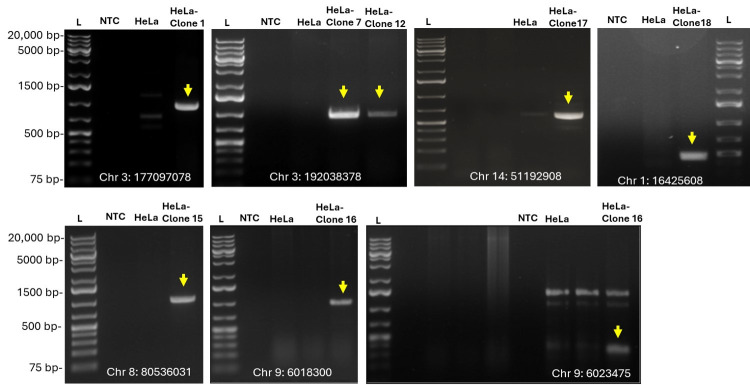
PCR validation of AAV integration sites identified by sequencing. Results of nested PCR of 0.25 μg of genomic DNA from seven HeLa-AAV2-eGFP single clones confirmed AAV integration at the indicated chromosomes. NTC is no template control; L is 1 Kb plus DNA ladder. The arrow indicates an amplicon of the expected length.

The PCR-free amplification based on CRISPR-Cas9 cleavage for long-read nanopore sequencing workflow revealed unique AAV2-eGFP integration junctions distributed in seven HeLa-AAV2-eGFP single clones (Figure 3C and Figure S3A–G). Most identified integration sites were matched with those detected by probe hybridization capture short-read sequencing. However, an additional integration event on chromosome 9 at position 601830 in clone 16 was identified only by the CRISPR-Cas9-based approach and was not detected by the hybridization capture method. Bioinformatic analysis further annotated the nearest neighboring genes located near the insertion sites (Figure 3C). All predicted integration junctions were further validated by PCR and sequencing (Figure 4). These data revealed one insertion site in clones 1, 7, 12, 15, 17, and 18, while two independent integration sites were detected in clone 16.

Although the two complementary target enrichment methods and sequencing strategies differ in technical characteristics and performance, both yielded consistent integration site profiles of HeLa-AAV2-eGFP single-cell clones in this study. Collectively, these results confirm that most HeLa-AAV2-eGFP clones carry a single, well-defined integration site, precisely mapped by both methods. Moreover, the Cas9-mediated Nanopore approach is rapid, cost-effective, and broadly applicable for AAV genome integration analysis, making it suitable for assessing integration events in animal models or clinical trial samples. However, because all analyses were conducted in HeLa cells under in vitro conditions, the integration patterns observed here may not fully reflect those in primary human cells or in vivo animal and human tissues, and validation in these systems will be important.

## 4. Discussion

The rapid expansion of recombinant AAV vectors as gene therapy drugs has necessitated the development of more rigorous safety assessment tools, particularly for detecting genomic integration events. Here, we present two new tools: a novel set of cell clones with single AAV vector integrations and a PCR-free amplification based on CRISPR-Cas9 cleavage and long-read Nanopore sequencing that overcomes the technical bottlenecks of traditional integration site analysis. By comparing this novel workflow with a standard probe hybridization capture approach, we demonstrate that CRIPR-Cas9-based enrichment offers comparable accuracy with a significantly reduced processing time and complexity.

A primary challenge in analyzing AAV integration is the bias introduced by PCR amplification, which is required by most current methods, including LAM-PCR and short-read sequencing libraries. These biases are exacerbated by the complex secondary structures of AAV ITRs, which usually fail typical sequencing reactions. Our results indicate that the CRISPR-Cas9-based workflow effectively eliminates these amplification biases. By using Cas9 for targeted cleavage followed by direct adaptor ligation, we established a streamlined protocol that reduces the library preparation time from over 20 h (for hybridization capture) to approximately 5 h.

Furthermore, the integration of long-read Nanopore sequencing allowed us to span the entire junction between the vector and host genome. This capability is distinct from short-read Illumina sequencing, which requires fragmentation into small (~250 bp) pieces, potentially obscuring large structural variations or rearrangements often present at AAV insertion sites. In our single-cell HeLa cell models, both methods successfully identified the baseline integration sites, validating the reliability of the Cas9-Nanopore approach. The data identified a single insertion site in clones 1, 7, 12, 15, 17, and 18, whereas clone 16 exhibited two independent integration sites. In most clones, a single integration site was associated with one vector copy number of WPRE and eGFP. However, clone 1 showed a WPRE:eGFP copy number ratio of 2:1 despite the presence of only one mapped integration site. In contrast, clone 16 displayed one copy of WPRE and eGFP, yet two integration sites were detected. In these clones, PCR analysis confirmed the presence of the expected integration fragment at the mapped junction; however, it does not provide information on the internal structure of the integrated vector. The imbalance between WPRE and eGFP copy numbers, as well as the inconsistency between vector copy number and the number of integration sites, suggests structural rearrangement of the integrated AAV genome, which may involve WPRE duplication, partial deletion of the eGFP region, concatemer formation, or other recombination events. Notably, the CRISPR-Cas9-based workflow also enabled detection of AAV integration events in mixed-cell populations, with insertion sites observed across multiple human chromosomes, suggesting potential utility in more complex sample types. In addition, the CRISPR-Cas9-based workflow provided the added advantage of preserving native DNA modifications and structural integrity.

An important limitation of this study relates to the sensitivity and limit of detection of the workflow. While the combined CRISPR-Cas9-based enrichment and long-read sequencing approach enabled mapping of integration sites in single-cell clones, its ability to detect low-frequency events in animal models or clinical trial samples has not been fully established. Detection sensitivity may be influenced by DNA input, enrichment efficiency, and sequencing depth. Additional studies will be required to define detection limits and further optimize the assay.

In conclusion, this study establishes CRISPR-Cas9-based enrichment combined with long-read sequencing as a powerful alternative to traditional hybridization-based methods. It offers a rapid, cost-effective, and unbiased solution for mapping AAV integration. As gene therapy applications continue to grow, this tool will be valuable for researchers aiming to comprehensively characterize vector–host interactions and ensure the safety of therapeutic interventions.

## Data Availability

All relevant data are included in this article and its Supplementary Materials. The raw library sequence data have been deposited in the NCBI Sequence Read Archive under BioProject accession number PRJNA1378741. Additional data are available from the corresponding author upon reasonable request.

## Supplementary Materials

The following supporting information can be downloaded at https://www.mdpi.com/article/10.3390/v18030315/s1, Figure S1: Evaluation of eGFP expression, and vector copy number in HeLa-AAV2-eGFP cells at 6 weeks post-transduction; Figure S2: Workflow comparison of target enrichment methods with an estimated processing time; Figure S3: Validation of AAV2-eGFP insertion sites in HeLa-AAV2-eGFP single clones; Table S1: List of primers and PCR condition used to validate AAV integration site; Table S2: sgRNAs targeting the AAV2-CAG-eGFP vector sequence.
