# Supplementary material for: Development of a Novel Method to Detect AAV Vector Integration
Source: Viruses. 2026 Mar 3;18(3):315. doi: 10.3390/v18030315 (PMC13030484; doi:10.3390/v18030315)
Supplement: Supplementary file 1 [file viruses-18-00315-s001.zip › viruses-4091771-supplementary.pdf]

Supplementary Materials

Supplemental Figure S1

A

Cell line	eGFP+ (%)
HeLa-Negative	0 %
HeLa-AAV2-eGFP	25.1 %
HeLa-AAV2-eGFP-BLM-treated	76.8 %

B

Cell line	WPRE VCN	eGFP VCN
HeLa-Negative	0.0038	0.0026
HeLa-AAV2-eGFP	0.3552	0.3528
HeLa-AAV2-eGFP-BLM-treated	1.1220	1.2869

Supplementary Figure S1 (related to Figure 1). Evaluation of eGFP expression, and vector copy number in HeLa-AAV2-eGFP cells at 6 weeks post transduction.

(A, B) Flow cytometry analysis of eGFP expression (A) and Vector copy number (VCN) quantification of WPRE and eGFP sequences in the mixture HeLa-AAV2-eGFP cells at 6 weeks post-transduction.

Supplemental Figure S2

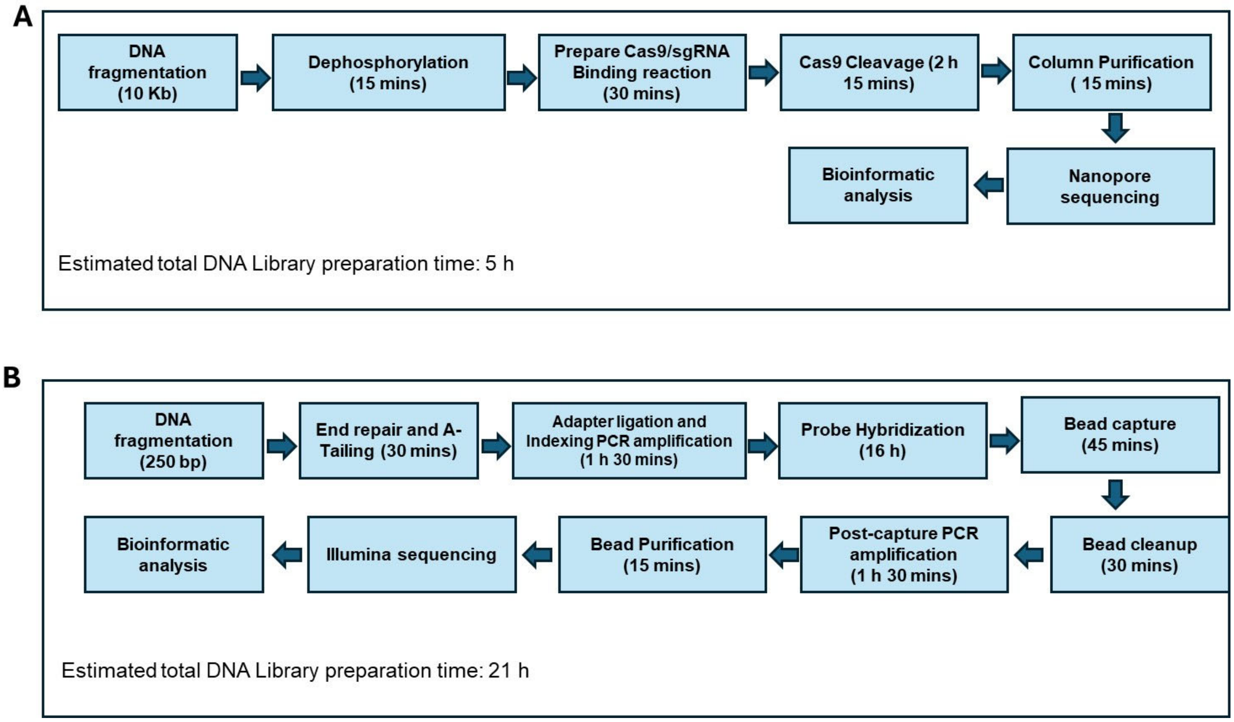

Supplementary Figure S2 (related to Figure 2). Workflow comparison of target enrichment methods with an estimated processing time.

(A) PCR-free amplification based on CRISPR-Cas9 cleavage workflow for long-read Nanopore sequencing.

(B) Probe Hybridization capture workflow for short-read Illumina sequencing.

Supplemental Figure S3

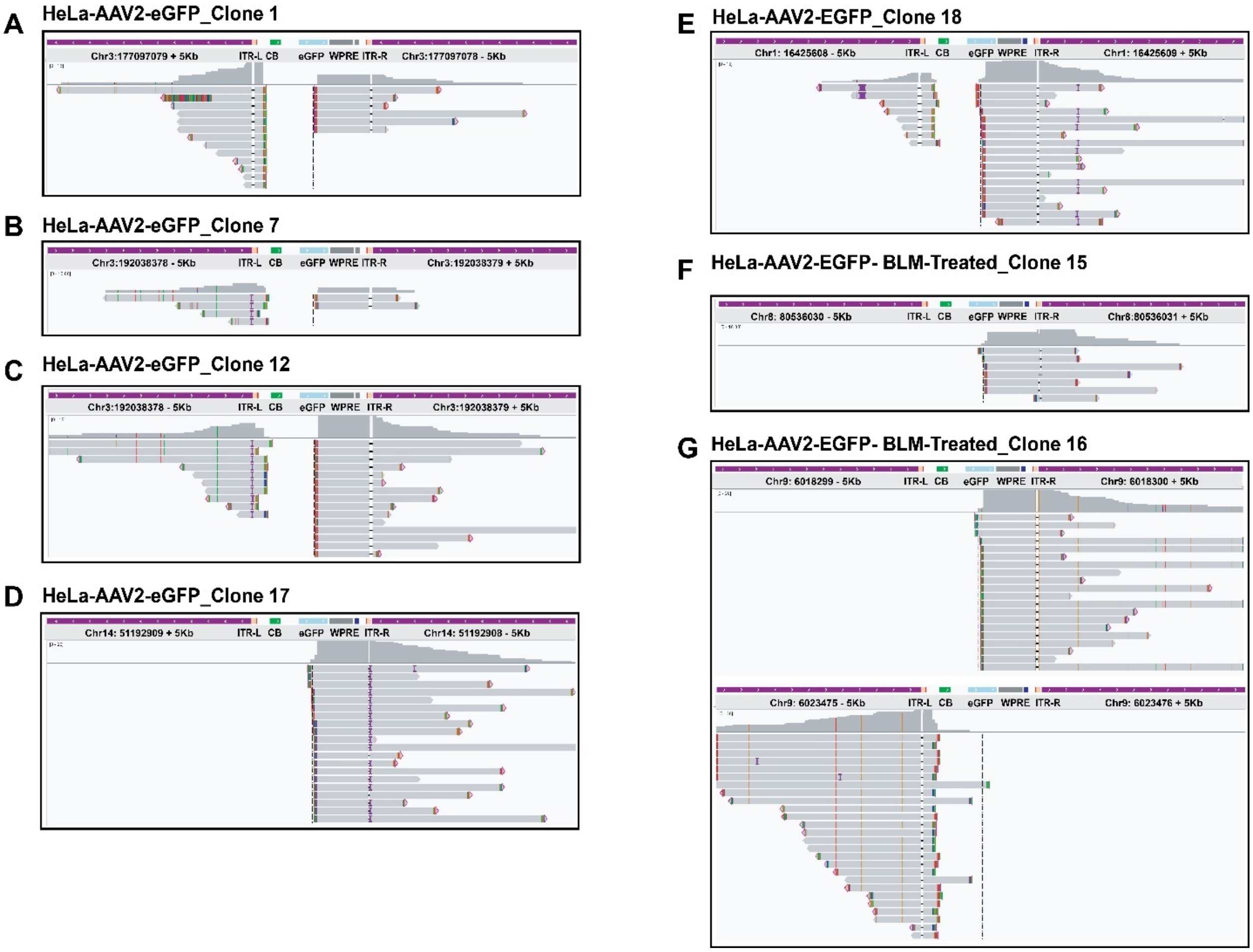

Supplementary Figure S3 (related to Figure 3). Validation of AAV2-eGFP insertion sites in HeLa-AAV2-eGFP single clones. (A-G) Integrative Genomics Viewer (IGV) snapshots showing Nanopore reads from individual HeLa-AAV2-eGFP clones, aligned to a custom chimeric reference consisting of ±5 kb of flanking human chromosomal sequence with the AAV2-eGFP vector cassette inserted at the integration breakpoint. The top track shows the positions of vector genetic elements (5' and 3' ITRs, promoter, EGFP and WPRE) within rAAV; bottom track shows long-read alignments at the left and right human-vector junctions. Gray bars represent individual read alignment, and colored ticks within reads denote single-nucleotide mismatches. Because library preparation used Cas9/gRNA-directed cleavage within the AAV2-eGFP vector (sgRNAs are listed in Supplemental Table 2), reads covering the left and right human-vector junctions originate from different reads and therefore do not span the entire vector insert.

Supplemental Table S1. List of primers and PCR condition used to validate AAV integration site.

	Name	Primer sequence (5' - 3')	Amplification length (bp)
HeLa-AAV2-GFP_Clone 1<br>Chr3: 177097078	Forward - 1	GTGTTGCCACCTGGATTCT	968
	Reverse - 1	GCCAAGAGCAGATACTCCAAATA	
HeLa-AAV2-GFP_Clone 7<br>Chr3: 192038378	Forward - 1	GTGTTGCCACCTGGATTCT	983
	Reverse - 1	GATATGGCCACACAGGTAGTTT	
HeLa-AAV2-GFP_Clone 12<br>Chr3: 192038378	Forward - 1	GTGTTGCCACCTGGATTCT	983
	Reverse - 1	GATATGGCCACACAGGTAGTTT	
Nested PCR 1<br>HeLa-AAV2-GFP_Clone 17<br>Chr14: 51192908	Forward - 1	GTGTTGCCACCTGGATTCT	1096
	Reverse - 1	GAGTTCTTGCCCTCGGATAAA	
Nested PCR 2<br>HeLa-AAV2-GFP_Clone 17<br>Chr14: 51192908	Forward - 2	GACGAGTCGGATCTCCCTT	600
	Reverse - 2	GGGTGTTACTACTCTACTCTGGT	
Nested PCR 1<br>HeLa-AAV2-GFP_Clone 18<br>Chr1: 16425609	Forward - 1	GTGTTGCCACCTGGATTCT	983
	Reverse - 1	CATACCTCTAATCCCAGCACTTT	
Nested PCR 2<br>HeLa-AAV2-GFP_Clone 18<br>Chr1: 16425608	Forward - 2	GACGAGTCGGATCTCCCTT	490
	Reverse - 2	CCTTCCTGGGTTCCTGAAGTA	
HeLa-AAV2-GFP_BLM-Treated_Clone 15<br>Chr8: 80536031	Forward - 1	GTGTTGCCACCTGGATTCT	975
	Reverse - 1	GACATGATGGTGTGTGCCTAT	
HeLa-AAV2-GFP_BLM-Treated_Clone 16<br>Chr9: 6018300	Forward - 1	GTGTTGCCACCTGGATTCT	981
	Reverse - 1	TAGGTCTGTGGTGGTGTATCA	
Nested PCR 1<br>HeLa-AAV2-GFP_BLM-Treated_Clone 16<br>Chr9: 6023475	Forward - 1	AAGGTCATGTACTGGGCATAAT	596
	Reverse - 1	GATCATCTCTGGAGATTGCTAGG	
Nested PCR 2<br>HeLa-AAV2-GFP_BLM-Treated_Clone 16<br>Chr9: 6023475	Forward - 2	TGGCGTTACTATGGGAACATAC	333
	Reverse - 2	TTCACTGCAGCCTTGACTT	

Supplemental Table S2. sgRNAs targeting the AAV2-CAG-eGFP vector sequence.

sgRNA	Position	Strand	Target sequence (5’ -3’)
sgRNA 1	78	-	CACTGAGGCCGGGCGACCAA
sgRNA 2	100	+	GAGTGGCCAACTCCATCACT
sgRNA 3	305	-	ACACTTGATGTACTGCCAAG
sgRNA 4	348	-	TTACCGTCATTGACGTCAAT
sgRNA 5	417	-	ACGTAGATGTACTGCCAAGT
sgRNA 6	1191	-	GACCAGGATGGGCACCACCC
sgRNA 7	1276	+	AAGTTCATTTGCACCACCGGC
sgRNA 8	1432	+	ACCATCTTCTTCAAGGACGA
sgRNA 9	1533	+	CAAGGAGGACGGCAACATCC
sgRNA 10	1581	+	AGCCACAACGTCTATATCA
